# Introducing Meta-Partition, a Useful Methodology to Explore Factors That Influence Ecological Effect Sizes

**DOI:** 10.1371/journal.pone.0158624

**Published:** 2016-07-13

**Authors:** Zaida Ortega, Javier Martín-Vallejo, Abraham Mencía, Maria Purificación Galindo-Villardón, Valentín Pérez-Mellado

**Affiliations:** 1 Department of Animal Biology, University of Salamanca, Salamanca, Spain; 2 Department of Statistics, University of Salamanca, Salamanca, Spain; Indiana University Bloomington, UNITED STATES

## Abstract

The study of the heterogeneity of effect sizes is a key aspect of ecological meta-analyses. Here we propose a meta-analytic methodology to study the influence of moderators in effect sizes by splitting heterogeneity: meta-partition. To introduce this methodology, we performed a meta-partition of published data about the traits that influence species sensitivity to habitat loss, that have been previously analyzed through meta-regression. Thus, here we aim to introduce meta-partition and to make an initial comparison with meta-regression. Meta-partition algorithm consists of three steps. Step 1 is to study the heterogeneity of effect sizes under the assumption of fixed effect model. If heterogeneity is found, we perform step 2, that is, to partition the heterogeneity by the moderator that minimizes heterogeneity within a subset while maximizing heterogeneity between subsets. Then, if effect sizes of the subset are still heterogeneous, we repeat step 1 and 2 until we reach final subsets. Finally, step 3 is to integrate effect sizes of final subsets, with fixed effect model if there is homogeneity, and with random effects model if there is heterogeneity. Results show that meta-partition is valuable to assess the importance of moderators in explaining heterogeneity of effect sizes, as well as to assess the directions of these relations and to detect possible interactions between moderators. With meta-partition we have been able to evaluate the importance of moderators in a more objective way than with meta-regression, and to visualize the complex relations that may exist between them. As ecological issues are often influenced by several factors interacting in complex ways, ranking the importance of possible moderators and detecting possible interactions would make meta-partition a useful exploration tool for ecological meta-analyses.

## Introduction

Meta-analysis is a quantitative methodology to analyze results from different studies with the aim of integrating results in a common general conclusion [1–3]. In ecology, meta-analyses are important contributors of effective thinking, since they address the strength of ecological patterns and hypotheses more than solely deal with p-values [4, 5]. In ecological meta-analyses, however, it is usually more important to explain heterogeneity than to integrate results [4, 6].

There are mainly two sources of variability that can explain the heterogeneity of the effect sizes included in a meta-analysis: (1) the sampling error variability, and (2) the between-studies variability, which may be due to true heterogeneity among a population of effect sizes due to biological factors, or it may be due to variations in the study design [6]. We are interested in this second type of variability of effect sizes, which is usually referred as heterogeneity of effect sizes [6]. The mean amount of heterogeneity explained by causal factors of interest in ecological meta-analyses is smaller than in other fields of study, mainly because of the complexity of relationships between different environmental, physiological or genetic factors [5, 7]. However, variability is a key aspect of ecology, so it is important to improve meta-analytic methodologies that deal with heterogeneity of ecological effect sizes [4, 8, 9].

There are meta-analyses that model the contribution of continuous co-variables to heterogeneity of ecological effect sizes, usually know as meta-regressions [9–11]. We present here a methodology of meta-analysis with algorithmic partition of heterogeneity by categorical or quantitative moderators [12]. As for meta-regression, its advantage is that it allows to identify main factors influencing the variability of a given effect size. The general approach of our methodology is analogous to Classification and Regression Tree Analysis (CART). This technique is an algorithmic partitioning method to be used both for regression and classification. The goal is to produce subsets of the data; which are as homogenous as possible with respect to a target variable [13]. In meta-analysis the target variable is the effect size. The CART method has been extensively used for ecological data (see, for example, [14, 15]). We refer here to it as meta-partition for the analogy, and this is the first paper about meta-partition in any field of Biology. To test and introduce this new methodology, we analyzed previously published data of a meta-analysis (specifically, meta-regression) about the relation between biological traits of wetland vertebrates and species sensitivity to habitat loss [16]. The use of previously published data allows us to compare meta-partition with meta-regression. Among other published meta-regressions, we have selected this study [16] because it deals with a big sample size of effect sizes (n = 334), because it uses several moderators that are suitable for meta-partition, and because their data about effect sizes and moderators are freely available online.

As habitat loss is the main thread to wildlife, it is important to study which traits of species are related to their sensitivity to habitat loss (see [16], and references therein). The study focuses in the relation between mobility and reproductive rate of wetland vertebrates with sensitivity of wetland habitat loss [16]. Sensitivity to habitat loss would be the relation between population abundance and the amount of wetland in a landscape. A detailed review about the existing hypothesis and the existing knowledge about the influence of mobility and reproductive rates in sensitivity to habitat loss is provided in the paper [16]. To assess if animal mobility and reproductive rates would influence their sensitivity to wetland loss, the authors conduct meta-regressions of the published data of 334 populations of wetland vertebrates (mammals, birds, reptiles and amphibians, [16]). Here we re-analyze the same data [16] with meta-partition in order to compare both methodologies.

## Materials and Methods

### Methodology of meta-partition

There are two approaches based on distinct assumptions under which the researcher may integrate the effect sizes. The first approach, the fixed effect model, considers that all estimators of the effect size estimate a single parameter and, so, it takes the recovered studies as the only items of interest. In contrast, the random effects model assumes that the studies are a random sample from some hypothetical population of studies.

In meta-analysis, let θ denotes the value of the chosen measure of the effect (difference of means, odds-ratio, correlation coefficient, difference of proportions etc.) which may be estimated from the different studies. Denote, ${\hat{\theta }}_{1}\ldots {\hat{\theta }}_{k}$ the estimates from these *k* retrieved studies and consider the hypothesis:

$$H_{0}:(\theta_{1}=\;\ldots \;=\;\theta_{j}\;=\;\ldots \;=\;\theta_{k}=\;\theta$$ (1)

An essential assumption in meta-analysis is the homogeneity of the treatment effect across all studies. To this purpose, we test the hypothesis of a common effect size fitting a fixed effect model. Fixed effect model assumes that experimental treatment effects vary across studies only from random error, so it is assumed that variation across studies is homogeneous [1, 17]. Therefore, a test of this hypothesis may be based on the magnitude of the statistic:

$$Q_{H}=\;\sum_{i=1}^{k}{\left({\hat{\theta }}_{i}-\;\hat{\theta }\right)}^{2}\;\cdot \;w_{i}$$ (2)

This statistic follows a χ^2^ distribution with (*k-1*) degrees of freedom. In addition, $\hat{\theta }$ is the overall effect size and *w*_*i*_ are given by:

$$w_{i}=\;\frac{1}{{\hat{\sigma }}_{{\hat{\theta }}_{i}}^{2}}$$ (3)

The random effects model considers that the studies as a random sample from a superpopulation of studies with mean θ and variance τ^2^. This model includes a study variation because each study would have its own underlying effect size. Furthermore, as before, suppose that the estimates ${\hat{\theta }}_{i}$ have a normal distribution, $N(\theta_{i};\;\sigma_{{\hat{\theta }}_{i}})$. Therefore, the estimator of the variability has two components: the between studies and the within studies variance:

$${\hat{\theta }}_{i}\;\equiv N\;\left(\theta_{i};\;\sqrt{\sigma_{{\hat{\theta }}_{i}}^{2}+\;\tau^{2}}\right)$$ (4)

In this model the test of homogeneity will test the null hypothesis, *H*_0_: *τ*^2^ = 0.

In meta-regression a set of variables is defined as explanatory variables of heterogeneity. The expression of the model under the assumption of fixed model is:

y=Xβ+ ε (5)

Where **y** is the column vector of the effect sizes, *X* is a matrix that contains the values of the explanatory variables, *β* is a column vector of regression coefficients and ε is a vector of errors.

The mixed model is a random-effects regression approach that may explain additional heterogeneity due to external variables. In this approach we assume that:

$$y_{i}=\;{\hat{\theta }}_{i}+\;\varepsilon_{i},\text{ where }\varepsilon_{i}\;\equiv N\left(0;\;\sigma_{i}\right)$$ (6)

and,

$$\theta_{i}=X_{i}\alpha +\;\delta_{i},\text{ where }\delta_{i}\;\equiv N\left(0;\;\tau \right)$$ (7)

Where α is a column vector of random-regression coefficients and *δ*_*i*_ represents the random effect for the ith trial having the same covariate values. So, the general model is given by:

Y=Xα+ δ+ ε (8)

Considering that *δ*_*i*_ and *ε*_*i*_ are independent, then

$$var\;\left(y_{i}\right)=\;\sigma_{i}^{2}+\;\tau$$ (9)

Meta-partition is proposed under the fixed model assumption. This procedure regards that one or several external variables gives rise to such heterogeneity. Meta-regression models are largely used to determine the average effect of an independent variable on effect sizes. Thus, when interventions are developed from regression model results, they are geared toward the average member of the population, without consideration of special needs of population subgroups. Regression modeling does allow for the testing of statistical interactions among independent variables, which assess differences in the effects of one or more independent variable according to levels of another independent variable. However, statistical interactions can be difficult to interpret, particularly when three or more variables are assessed at a time. Meta-partition is a recursive procedure that identifies mutually exclusive and exhaustive subgroup of a population of effect sizes.

**Fig 1:**
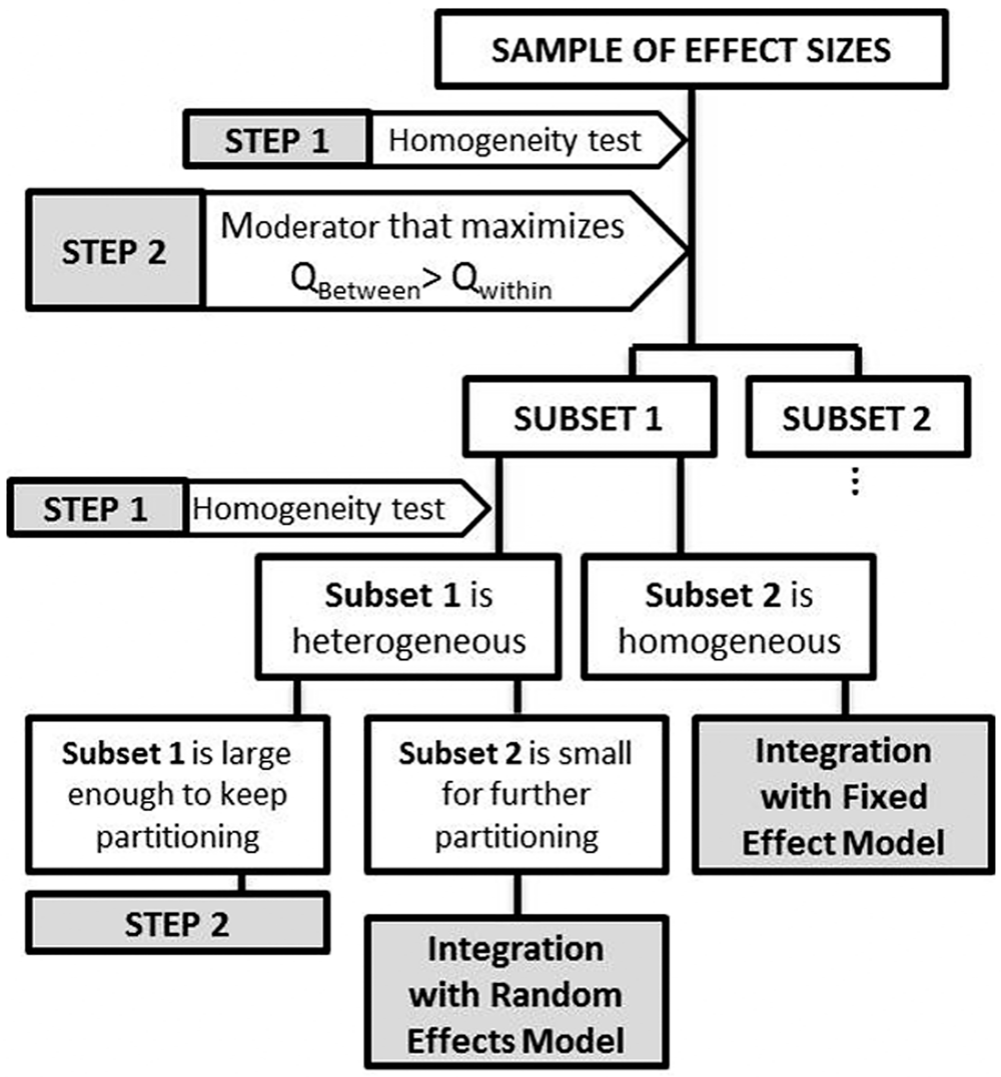
Diagram summarizing the methodology of meta-partition. Step 1 involves assessment of heterogeneity with the homogeneity test and the value of *I*^*2*^, step 2 involves selecting the best moderator according to Logworth value with p-value correction, and step 3 involves integration of effect sizes in final subsets.

Meta-partition is a method to be used with meta-analysis for explaining heterogeneity of effect sizes with moderators. Thus, we consider two types of variables: (1) quantitative response variables that provide effect sizes, and (2) the factors that are influencing effect sizes, or moderators, which may be qualitative or quantitative variables. Both, effect sizes and moderators must be selected *a priori* in order to avoid spurious relationships and the ecological fallacy [18, 19]. The approach consists in assessing heterogeneity of a sample of effect sizes and partitioning this variability with an algorithm that finds the best moderator of each partition. The best moderator is the one that explains the largest amount of variability between the potential subsets on each partition (see Fig 1).

Therefore, step 1 is to test the initial hypothesis of one real effect size with the fixed effect model, for testing homogeneity. If the set of effect sizes showed a significant heterogeneity, we then calculated the amount of heterogeneity with *I*^*2*^ [20, 21]. Afterwards, we will try to explain this heterogeneity by the moderators that were established *a priori* in the design of meta-analysis. Then, step 2 will be repeated until we reach one of these three situations: (1) to reach a subset of effect sizes that is homogeneous under the fixed effect model, (2) to reach a subset of effect sizes that is still heterogeneous but that it is too small to keep partitioning, or (3) to reach a subset of effect sizes that is still heterogeneous but the considered moderators are not able to explain the heterogeneity in this subset. Thus, if we reach a subset of effect sizes that is homogeneous, we will integrate the underlying common effect size by the fixed effect model. Otherwise, when we reach a subset of effect sizes that is still heterogeneous but is not suitable for further partition, we will integrate its effect sizes by the random effects model (Fig 1). If none of these two situations is attained, we will keep partitioning each subset by the best moderator of heterogeneity until one of them is reached (Fig 1). Anyway, integration of effect sizes is not the key point in meta-partition, since its strength is to deal with heterogeneity.

#### Step 1: study of heterogeneity

The first step of meta-partition is to test if there is a significant heterogeneity that could be attributed to ecological or methodological moderators. To this purpose, we test the hypothesis of a common effect size fitting a fixed effect model. Fixed effect model assumes that experimental treatment effects vary across studies only from random error, so it is assumed that variation across studies is homogeneous [1, 20]. *Q*_*H*_ is used to test the homogeneity. The null hypothesis of this test is that every sample effect size estimates a unique population effect size, H: (θ1 = … = θ i = … = θ k = θ). However, this test of homogeneity has low power [22] and not significant results do not imply that true homogeneity exists. Thus, Jeng, Scott & Burmeister [23] regard that p-values less than 0.10 suggest heterogeneity. Therefore, the magnitude of the heterogeneity is also assessed by *I*^*2*^ coefficient [21, 24]. It is calculated as:

$$I^{2}=\;\frac{\frac{Q_{H}}{k-1}-1}{\frac{Q_{H}}{k-1}},$$ (10)

where *Q*_*H*_ is Cochran’s Q, and (*k-1*) are its degrees of freedom. This coefficient represents the proportion of total variance that is explained by the differences between studies over the variance that would be attributed to chance. *I*^*2*^ is an effect size of variability, so it changes the focus of heterogeneity from significance of p-values to a real measure of variability [4].

#### Step 2: partition analysis

If the whole sample of the effect sizes (or, in subsequent iterations, the corresponding subset of effect sizes) is heterogeneous, we will proceed with the second step of meta-partition. Second step consists of dividing the subset of effect sizes by the moderator that explains the largest amount of variability between resulting subsets in relation with variability within each of the potential subsets (Fig 1). Thus, a partition method is proposed to explore the importance of each moderator on explaining heterogeneity. This partition is based on classification and regression trees (CART, see [13]) and the procedure to incorporate data structure in meta-analysis proposed by Hedges & Olkin [1]. The method starts from a set of M effect sizes and a set of moderators (X_1_, X_2_, …, X_p_) defined *a priori* in the design of the meta-analysis. We suppose that these moderators are associated to effect sizes: they could be ecologically relevant moderators, or they could be moderators related to the quality of design of analyzed studies that may provide sensitivity analysis [25]. The aim of the algorithm of partition is to find r disjoint classes (m_1_, m_2_…m_r_) from the set of effect sizes such that the classes will be the most homogeneous within them, while being the most heterogeneous between them. The technique involves the partition of the sum of squares from the test of homogeneity into two components [1]:

$$Q_{H}=\;Q_{B}+\;Q_{W},$$ (11)

in where:

$$Q_{B}=\;\sum_{i=1}^{r}{\left({\hat{\theta }}_{i}\;-\;\hat{\theta }\right)}^{2}\cdot w_{i}$$ (12)

$$Q_{w}=\;\sum_{i=1}^{r}\sum_{j=1}^{k}{\left({\hat{\theta }}_{ij}\;-\;\hat{\theta }\right)}^{2}\cdot w_{ij}$$ (13)

Thus, ${\hat{\theta }}_{ij}$ is the effect size in the -jth study in the -ith class, ${\hat{\theta }}_{i}$ the pooled effect size in the -ith class and $\hat{\theta }$ the pooled effect size in the total set of studies. When the null hypothesis of common effect is true, the value of the between-class fit statistic (*Q*_*B*_) and the within-class fit statistic (*Q*_*W*_) will follow a *χ*^*2*^ distribution with (*p-1*) and (*k-p*) degrees of freedom, respectively. In each partition, this method will minimize the intraclass distance (*Q*_*W*_) and so maximize the interclass distance (*Q*_*B*_). An indicator of importance of the each factor is the ratio of both distances.

The algorithm of this procedure is: (1) Looking for the lowest number of classes for each moderator that maximizes *Q*_*B*_ value (usually two). The combination of levels of the moderator will depend on the type of moderator: if it is nominal, all possible ways to combine the categories would need to be evaluated; if it is ordinal, the combination of categories must preserve the order in the data; and, if the moderator is continuous, the algorithm finds the value that maximizes the interclass distance, and splits the moderator into two groups regarding this value. (2) The first partition is chosen to maximize the difference in the response (effect size) between the levels of the moderator. There are two criteria to do the partition: the p-values of *Q*_*B*_ or the ratio (*Q*_*B*_/(*p-1*))/(*Q*_*W*_/(*k-p*)). Sometimes, *Q*_*B*_ value is not statistically significant but the ratio is large, then the meta-analysist can choose this criterion to split in each partition. Ideally, the researcher would select the combinations of levels that make biological sense based in scientific evidences on the research question. If there scientific evidences were lacking, the researcher could apply this combination of criteria in order to have an objective rule for partition. (3) Testing if the classes obtained in the first partition are homogeneous using *Q*_*H*_ and *I*^*2*^. (4) If any class is heterogeneous, then the step 2 will be repeated with the rest of moderators. (5) The partition process will stop when all classes are homogeneous, when they are final due to small sample size, or if none of the moderator explains the heterogeneity (see above). Regarding the criterion to consider small sample size, to stop partitioning, there is great controversy about the minimum number of studies that must present a meta-analysis. Davey et al. [26] analyzed the characteristics of 22.453 meta-analyses from the Cochrane Database of Systematic Reviews, and found that the median number of studies of a meta-analysis was 3 (inter-quartile range: 2–6). Here, we have considered that 8 would be a suitable minimum number of studies to stop partitioning as a general rule, in order to avoid confounding factors, but the researcher must follow its own criterion, and continue partitioning for situations when it would make biological sense.

Over-fitting is a limitation of this kind of procedures [15]. This limitation, in meta-analysis, is mainly due to: (1) to the large number of comparisons that are necessary to create the partition trees, (2) to the use of tests of homogeneity to do the meta-analysis and (3) sample size in each partition. To avoid the problem related with multiple comparisons, we use an approach based in Montecarlo calibrations [27] that is implemented in JMP software. This approach controls for type I error and is less conservative than the Bonferroni correction. Hardy and Thompson [28] discussed the problems that would arise with the use of the *Q*_*H*_ test depending on the number of studies that are being integrated. To avoid this problem, we propose the use of several criteria described above to do each partition, and it is also important to set a minimun sample size to carry on each partition. Therefore, this meta-analytical procedure should not be automatic, but rather each partition must be examined by the researcher. There would be two parts of the analysis where the critical evaluation of the quantitative results by the researcher would be valuable in order to decide about the homogeneity of the subsets. The first one would be after each partition, assessing the significance level and the ratio *Q*_*B*_/*Q*_*w*_. The second one would be for each subset resulting of the partition, studying the values of *Q*_*H*_ and *I*^*2*^.

#### Step 3: integrating effect sizes

When a final subset is homogeneous, we integrate its common effect size with the fixed effect model, as explained above. However, if the heterogeneity between effect sizes is not explained by the fixed effect model and the subset is no longer divisible due to small sample size, the random effects model will be used to integrate its common effect size. Under this assumption, the weights are calculated by:

$$w_{i}=\;1/\left({\hat{\sigma }}_{{\hat{\theta }}_{i}}^{2}+\;{\hat{\tau }}^{2}\right),$$ (14)

in where σ^2^ is the estimated variance of the effect sizes of the subset, and *τ*^2^ is given by the DerSimonian-Laird estimator [29]:

$${\hat{\tau }}^{2}=\;\frac{Q_{H}-\left(k-1\right)}{\sum_{i=1}^{k}w_{i}-\;\frac{\sum_{i=1}^{k}w_{i}^{2}}{\sum_{i=1}^{k}w_{i}}},$$ (15)

where *k* is the number of studies, *Q*_*H*_ is the homogeneity test and *w*_*i*_ is the weight under the fixed effect model.

#### Statistical software

The partition procedure has been carried on with JMP-10 (SAS Institute, Inc., Cary, NC, USA) and Metawin 2.0 [30].

### Example: a meta-partition of data about species sensitivity to habitat loss of Quesnelle et al. (2014)

#### Description of the data

To test the new methodology, we performed a meta-partition with the data used in published meta-regressions about the relation between biological traits of wetland vertebrates and species sensitivity to habitat loss [16]. Data extraction and filtration is detailed explained in the paper [16], including a PRISMA diagram of the filtration process. The effect size of interest is the correlation between the amount of wetland in a landscape and the population abundance of a given wetland population, transforming Pearson correlation coefficients into Fisher’s z-scale effect sizes. Seven moderators are considered to possibly affect the effect sizes, three of them related to the design of the studies and four of them related to biological traits of the wetland species [16].

The three moderators related to the study are: (1) the measurement method of the *amount of wetland*, with two categories, *amount-based studies* and the *configuration-based studies*, (2) the relation between *sampling effort* and the size of wetlands, with three categories, *area-independent* (sampling effort was consistent among sampled wetlands), *area-dependent* (sampling effort increased in proportion to the wetland area), and *unknown* (sampling effort was unknown), and (3) if the *sampled wetland was included* in the calculation of wetland area or not, with two categories, *included* and *not included* (see all details in [16]).

The four moderators related to biological traits of species are: (1) *taxon*, with four categories, *mammals*, *birds*, *reptiles*, and *amphibians*, (2) *home range* area, a continuous moderator expressed in ha, (3) *body size*, a continuous moderator expressed in cm, and (4) the *reproductive rate* of species, a continuous moderator expressed as the mean litter or clutch size multiplied by the mean number of litters or clutches per year. *Home range* and *body size* are indicators of the mobility ability of species, sometimes related to the ability of dispersion and colonization of new habitats (see a detailed explanation in [16]). All details about these moderators and their extraction from literature are provided in the paper [16].

We downloaded all data from Table A and Table B of the Supporting Information section of the online link of the publication (http://journals.plos.org/plosone/article?id=10.1371/journal.pone.0090926#s6).

#### Description of the meta-partition

We have performed a meta-partition analysis on the data extracted by Quesnelle et al. [16]. We have used the sample of effect sizes that include all information about moderators, a dataset of 334 effect sizes (see Table S1 and Table S2 in [16]). Our meta-partition includes all the seven moderators, the three related with the design of the study and the four biological moderators. A detailed explanation about all moderators can be found in the paper [16].

**Fig 2:**
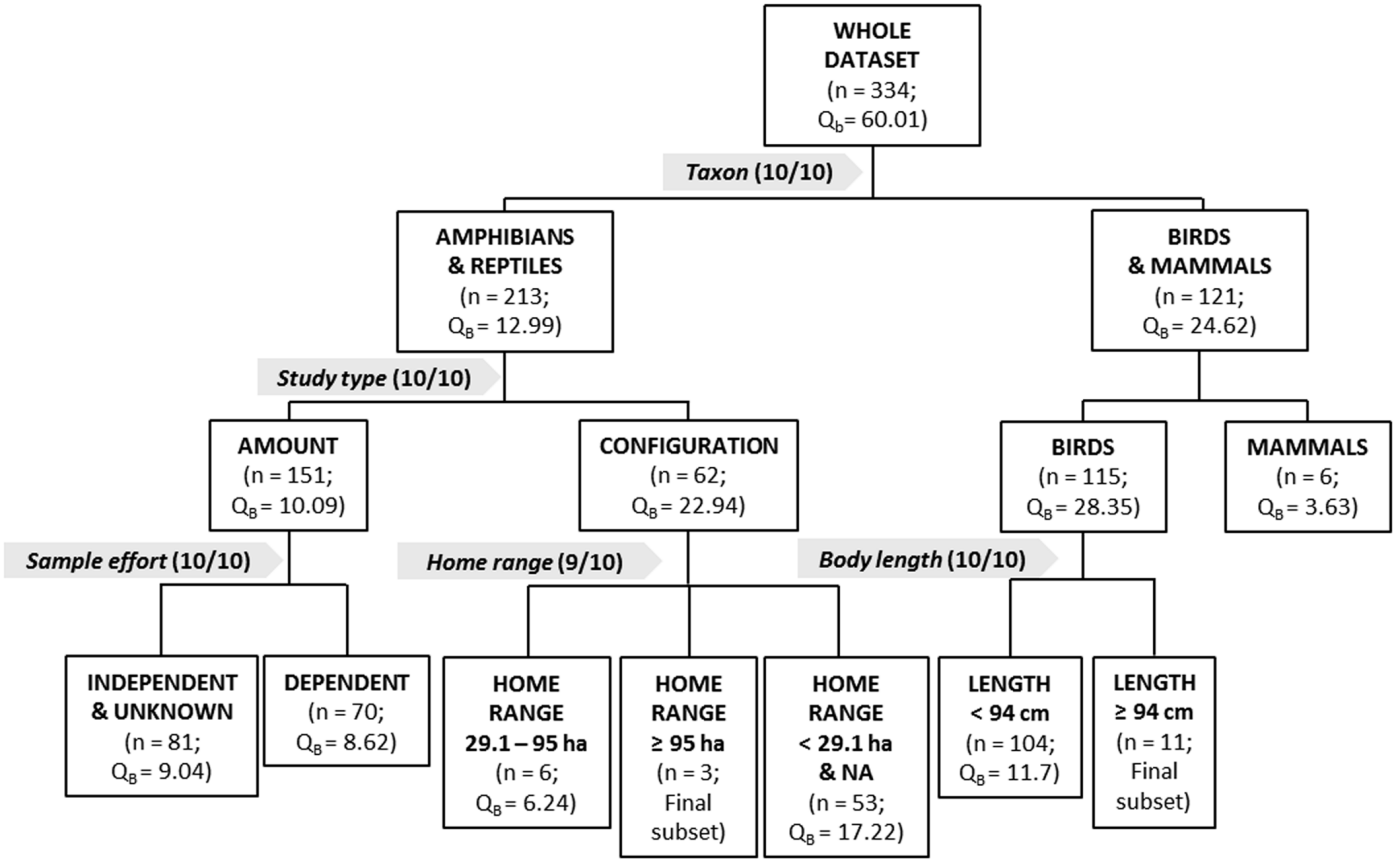
Summary tree of the meta-partition of the main traits influencing sensitivity to wetland habitat loss. Within each partition, effect sizes are organized from left (smaller) to right (larger). The best moderator that explains the variability of each partition is highlighted with a grey arrow, and numbers in brackets mean (in the form of *X*/*Y*, e.g. 9/10) the number of times this partition is kept (that is, is similar than in the original tree) in the sensitivity analysis (*X*) in comparison with the number of times that a partition is replicated (*Y*). The complete partition trees are provided in the S1 Appendix.

In order to test the stability of the meta-partition trees and the potential limitation of over-fitting, we performed a sensitivity analysis: we replicated the tree 10 times, randomly eliminating the 5% of the effect sizes for each replication. The procedure of meta-partition was repeated for each of the samples with the same protocol conducted for the global sample. If the moderators and the number of final subsets of a replication were the same as the original tree, then it was counted, and if they were different, it was not counted. In other words, we provided each partition of the trees with a measure of *X*/10, where *X* means the number of times that this partition was similar than the original tree. These values are provided in the partition trees (see Fig 2, Fig A in S1 Appendix, and Fig B in S1 Appendix).

## Results

The initial dataset of the effect sizes of 334 populations is highly heterogeneous (*Q*_*H*_ = 711.50; p < 0.0001). Thus, we start partitioning the initial subset, searching for the moderator that minimizes heterogeneity within subsets that leads, while maximizes the heterogeneity including. We show the process of first partition in detail in order to illustrate the method, and subsequent summarized partitioning results could be found in Table A in S1 Appendix.

First, we search, among all candidate moderators to partition, the combination of levels that maximizes the heterogeneity between these subsets to be made by the potential partition. In order to do this, we calculated the *Q*_*B*_, the ratio (*Q*_*B*_/(*p-1*)/*Q*_*W*_/(*k-p*)) and the p-value (see Materials and Methods) for all possible combinations of the levels of all candidate moderators. As an example, results for the moderator *taxon* are reported in Table 1.

**Table 1:** Results of the heterogeneity of the possible combinations of levels of the moderator *taxon*. The selected combination is marked in bold.

Combinations	Q_B_	(Q_B_/(p-1)/Q_W_/(k-p))	Logworth*
“amphibians”–“reptiles + mammals + birds”	52.86	26.65	6.52
“amphibians”–“reptiles + birds + mammals”	0.92	0.43	0.27
“birds”–“amphibians + reptiles + mammals”	32.6	15.94	4.10
“mammals”–“amphibians + birds + reptiles”	51.34	25.82	6.33
“amphibians + birds”–“reptiles + mammals”	11.92	5.66	1.70
“amphinians + mammals”–“birds + reptiles”	23.02	11.1	2.99
**“amphibians + reptiles”–“birds + mammals”**	**68.01**	**35.09**	**8.12**

* Logworth = -log10(p-value)

The largest heterogeneity of effect sizes is found between the combination “Amphibians and Reptiles” and “Birds and Mammals”. Thus, for the moderator *taxon*, the subsets resulting of the partition would be: “Amphibians and Reptiles” and “Birds and Mammals” (Table 1). We repeated this operation with the other moderators, selecting for each of them the combination of levels that maximized the heterogeneity among subsets. Then, we selected among the candidate moderators to partition, the one that maximized the heterogeneity of effect sizes between subsets of the combination of levels that, at the same time, maximized the heterogeneity of effect sizes between formed subsets (Table 2).

**Table 2:** Results of the heterogeneity of the different candidate moderators to perform the first partition. The selected moderator is marked in bold.

Moderator	Q_B_	Logworth* of Q_B_	Q_W_	(Q_B_/(p-1)/Q_W_/(k-p))	Cutpoint
**Taxa**	**68.01**	**8.12**	**643.50**	**35.09**	**amphibians, reptiles**
Study Type	16.69	2.26	694.82	7.97	amount
Sampling Effort	18.75	2.20	692.76	8.99	unknown, independent
Patch Area	0.36	0.15	711.15	0.17	Yes
Lenght (cm)	60.38	7.10	651.13	30.79	32.00
Repro	66.70	8.07	644.82	34.34	27.20
Home Range (ha)	31.66	3.03	679.86	15.46	0.29

* Logworth = -log10(p-value)

Thus, the main moderator that affects sensitivity to wetland habitat loss is *taxon*, with amphibians and reptiles on one subset and birds and mammals on the other (Fig 2). Effect sizes are smaller for amphibians and reptiles (mean *z* = 0.074, sd = 1.45) than for birds and mammals (mean *z* = 0.210, sd = 1.28).

Among amphibians and reptiles, the main moderator that affects effect sizes is *study type*, with herpetofauna studied by *amount* (area-based buffers) on one side (mean *z* = 0.058, sd = 1.37, n = 151) and herpetofauna studied by *configuration* (nearest-neighbour distances or incidence function model) on the other (mean *z* = 0.132, sd = 1.58, n = 62; Fig 2). Among amphibians and reptiles studied by *amount*, next main moderator affecting effect sizes is the *sampling effort*, with herpetofauna studied by *amount* with *sampling effort* being *independent* of effect sizes (or *unknown*) on one side (mean *z* = 0.037, sd = 1.33, n = 81) and herpetofauna studied by *amount* with *sampling effort* being *dependent* of effect sizes on the other (mean *z* = 0.100, sd = 1.36, n = 70). For both subsets, next main moderators about the sensitivity of habitat loss are the *reproductive rate*, and then both indicators of mobility (*home range* and *body length*). A detailed partition of subsequent subsets is provided in the Fig A in S1 Appendix. Within amphibians and reptiles studied by *configuration*, next main moderator that affects sensitivity to habitat loss is *home range*, where herpetofauna with middle *home ranges* (29.1–95 ha) show smaller sensitivity to habitat loss (mean *z* = -0.243, sd = 1.18, n = 6), herpetofauna with larger *home ranges* (≥ 95 ha) show middle sensitivity to habitat loss (z = 0.154, sd = 0.91, n = 3), and herpetofauna with smaller *home ranges* (< 29.1 ha) show higher sensitivity to habitat loss (mean *z* = 0.161, sd = 1.47, n = 53; Fig 2). For subsequent partitions, *reproductive rate* is the main moderator that affects effect sizes (see Fig A in S1 Appendix). Although confounding factors may arise when sample size of subsets is getting smaller, our data consistently show a general trend to interaction among *reproductive rate* and the moderators of mobility for amphibians and reptiles (Fig A in S1 Appendix).

Among birds and mammals, the main moderator is, again, the *taxon*, being birds less sensitive (mean *z* = 0.185, sd = 1.21, n = 115) to habitat loss than mammals (mean *z* = 0.408, sd = 0.94, n = 6). Within birds, the main moderator is *body length*, with the bigger birds (≥ 94 cm) being more sensitive to wetland loss (mean *z* = 0.511, sd = 0.98, n = 11) than the rest of birds (mean *z* = 0.165, sd = 1.12, n = 104). Among these birds that are smaller than 94 cm long, next main moderator is *reproductive rate*, being birds with a smaller reproductive rate (< 3.9) more sensitive to habitat loss (mean *z* = 0.288, sd = 1.33, n = 20) than birds with a larger (≥ 3.9) reproductive rate (mean *z* = 0.142, sd = 1.01, n = 84). After the *reproductive rate*, moderators about the design of the study and about mobility are differently affecting effect sizes into the different subsets (see Fig B in S1 Appendix).

Results of the sensitivity analysis of the meta-partition are reported in the trees (Fig 2, Fig A in S1 Appendix, and Fig B in S1 Appendix). For the first three partitions of each tree, almost 100% of the replications keep the same moderators as the original meta-partition tree. Certain instability arises in the last partitions of each tree, as it is expected, due to the decreases of effect sizes of each group. However, between 70% and 100% of the final partitions keep the same moderators as the original meta-partition tree. Regarding final subsets, 7 of the replications keep the same number of final subsets, 2 result in less final subsets (2 less final subsets), and 1 results in more final subsets (2 more final subsets) than the original meta-partition tree.

## Discussion

One of the main traditional limitations of meta-analysis is the heterogeneity, “mixing apples and oranges”, issue [8, 31, 32]. However, exploring the possible reasons for heterogeneity between studies is an important aspect of conducting a meta-analysis [33]. Gurevitch and Koricheva [34] stated that the analysis of heterogeneity in meta-analysis may be more important in ecology and evolution than in other disciplines. There are basically three approaches for modeling between-studies variation: fixed-, random, and mixed-effects models. In the fixed effect model, the estimated experimental treatment effects vary across studies only from random error. To assess homogeneity, heterogeneity is often tested. The random effects approach is an attempt to overcome the heterogeneity limitation. This model supposes the heterogeneity as unexplained and considers that the studies are a random sample from a superpopulation of studies with mean θ and variance τ^2^. However, this method is not considered a solution to the problem of heterogeneity [35, 36]. The random effects model relies on the unrealistic assumptions that the studies are representative of some hypothetical population of studies and the heterogeneity between studies can be represented by a single variance [37]. The mixed effects model could be the solution, since this approach includes additional random variation and takes into account different factors or variables that could explain the difference between studies. In this way, meta-regression provides the advantage of exploring associations between study characteristics and study outcomes.

Meta-partition should be applied under the fixed effect model, since the main aim of this technique is to explore the heterogeneity in the retrieved studies. Peto [38] and Thompson and Pocok [37] argue that it is better to analyze the results from the retrieved studies under a common hypothesis and avoid other ambitious inferential hypothesis, which are very difficult to test accurately. The fixed effect model invites the analysis of subsets within the full set of data and, therefore, the exploration of external factors which could be important in the explanation of results. A further consideration is that the random effects model gives greater relative weight to smaller studies in case that heterogeneity would be large [39]. This aspect could affect the structure of association, and studies with low sample size could be overrepresented. Although several authors [40] advise about the mistake of changing from the fixed effect model to the random effects model when the heterogeneity has not been explained, we propose to do it in those subgroups where the homogeneity test is significant. This rule is only chosen in order to include the heterogeneity that has not been explained in the calculation of effect sizes, and it is not related with the assumptions of the random effects model.

Thus, the main aim of meta-partition is to explore the combination of factors that influence the magnitude of the effect sizes of interest. This information could be more valuable planning future experimental studies than estimating the particular effect size in each subgroup. Jennions, Lortie and Koricheva [41] state that generating hypothesis is an important role of ecological meta-analyses. A key point of ecology is to explain the variability of organisms in relation with environmental factors (see, for example, [42]). In this regard, meta-partition is a very powerful methodology because it allows us to identify and arrange the influence of the environmental factors in an effect size. In general, there are so many environmental factors that influence organisms that the variability we can explain is reduced to approximately between 2 and 7% [7]. Hence, a methodology as meta-partition may be decisive as it allows us to assess the robustness of generalizations of primary findings. Meta-partition lets us know the order of importance of the proposed moderators affecting the studied ecological effect size and in what direction goes the general pattern. In this sense, Hillebrand & Gurevitch [11] discuss the importance of exploring the heterogeneity in meta-analyses as a tool for improving and standardise the material and methods of primary studies.

Moreover, one can use meta-partition, as with CART studies [43–45], to explore the influence of many candidate moderators on the effect size of interest, and then perform more specific analysis with those main moderators obtained from meta-partition. This use would be analogous to meta-regression, but using categorical environmental factors rather than quantitative variables [4, 46, 47]. Meta-regression presents some limitations: (1) it assumes that the associations between variables are lineal, (2) it does not commonly explore the interactions between moderators, which might be important to explain the heterogeneity of effect sizes, (3) results are sensible to the presence of outliers, and (4) results could be affected by the problem of collinearity [48]. However, meta-partition does not present any of these limitations. There is a parallelism between meta-partition and stepwise meta-regression, where predictors are also included one at a time in successive order. However, in stepwise linear regression the predictors still have a linear effect on the dependent variable, while extensions of stepwise procedures, including interaction effects, are typically limited to the inclusion of two-fold interactions, since the number of higher order interactions—that would have to be created simultaneously when starting the selection procedure—is too large. Furthermore, the main aim of meta-partition is to obtain homogeneous groups and not to assess the effect of each moderator in the variability of effect sizes, as it would be the case of the stepwise meta-regression. Anyway, both methodologies are compatible and would and their combination would reinforce the results of the meta-analysis to extract generalizations.

Finally, meta-partition would also be compatible with phylogenetic meta-analyses, weighting effect sizes by phylogenetic distances between studied organisms [25, 49], as is been calculated in meta-regression (see, for example, [16]). Furthermore, meta-partition could be a valuable tool to assess how the quality of primary studies would affect the magnitude of effect sizes. A formal assessment of quality of studies could be made using a measurement scale by defining a questionnaire from a set of methodological factors that may affect the quality of studies. Several judges should be chosen to assess the questionnaire in order to minimise errors [50]. A categorical quality variable will be generated from the quality scores and included in partition process.

Just as meta-regression, meta-partition shows some limitations. Firstly, a large number of studies should be retrieved to apply meta-partition. Besides, the more factors are used, the more number of studies is necessary. The achievement of spurious groups is another limitation. Peto [38] and Hardy and Thompson [28] advise caution about the interpretation of the results arising from subsets of studies because such subgroups may be over-interpreted. Thus, the hypothesis about heterogeneity or the potential explanatory factors should be established *a priori*, and the choice of factors should be justified from an experimental point of view. Another drawback appears when the explanatory variable is sometimes defined in meta-analysis as a mean or proportion of characteristics of individuals within studies, for example for body length and reproductive rate in our example of wetland vertebrates. The use of these kinds of variables can produce an aggregation bias, generally known as the ecological fallacy [18]. The association between effect sizes and the aggregated variable in a level of study can be detected, but this association may be different in a level of individuals. This effect is well-described for meta-regression [10, 19]. A limitation of meta-partition would be related with over-fitting. Therefore, it is important that the researcher supervises each partition, paying attention to the different criteria of partition and their biological value. In addition, the use of a correction of p-values for multiple paired comparisons is also recommended.

Regarding the example used to present meta-partition, we want to point out that the study of Quesnelle et al. [16] is an example of a good meta-analysis regarding its exhaustiveness, transparency, and our criticisms are only about some limitations of the methodology of meta-regressions to deal with heterogeneity when many moderators may influence it. The first common result between both meta-analyses is that the taxon affects the sensitivity of wetland vertebrates to habitat loss, being amphibians and reptiles more tolerant than birds and mammals (see Fig 3 of [16]). However, the advantage of meta-partition is that it allows us to affirm, based in empirical evidence, that the taxon is the most important moderator among the seven that were consider in the study, over the design of the study, or the reproduction rate or the mobility of those vertebrates. This result makes unsuitable other analysis about the studied moderators in which all vertebrates are mixed, as the simple and multiple meta-regressions conducted by Quesnelle et al. [16] before considering the taxon. Furthermore, when they consider the taxonomic class, they find a significant effect of the taxon in the effect size, but they do not have the statistical tools that may quantify this heterogeneity and make subsets regarding it. Other example of the same phenomenon of the practical differences between meta-partition and meta-regression is that Quesnelle et al. [16], analyzing the whole dataset together, find that the design of the study does not affect the effect sizes. Nonetheless, the study type is the most important moderator affecting the effect size of the published studies about the sensitivity to habitat loss in the herpetofauna of wetlands (Fig 2). Thus, the study type should not be ignored for amphibians and reptiles, and a methodological discussion may be done in this regard for ecologists that compare their results when their studies would be conducted using different approaches. These examples in the differences of the results found in the meta-regressions of Quesnelle et al. [16] and our results with meta-partition highlight the importance of establishing an objective methodology that can assess the relative importance of the moderators considered for meta-analysis and their general relationships. We think that here lies the main utility of meta-partition, which may add an empirical criterion for meta-analysts to decide which moderators need to be considered in further analysis into the different levels of each one.

The biological discussion of our results and its comparison with those from Quesnelle et al. [16] is beyond the scope of this study, as we focus here in showing meta-partition and the comparison with meta-regression regarding its applications for ecological meta-analyses. Even though, the complete results and some comments regarding its biological implications are provided in the S1 Appendix. In general, the main advantages of meta-partition in comparison with meta-regression, regarding the analysis of the same data, are that meta-partition has allowed us to: (1) objectively considering the importance of each moderator in any subset of the initial dataset, (2) establish the direction of the relation between each moderator and the effect size, (3) visualize possible interactions between moderators and their complex relationships. Meta-regression may be better for quantifying the relationship between a moderator and the effect size, but meta-partition would be a strong exploration tool that would improve the use of subsequent meta-analyses.

## Supporting Information

S1 Appendix Extended results of the meta-partition about the sensitivity of vertebrates to wetland habitat loss. It comprises the detailed trees of the meta-partition, with comments about the intensity and direction of the effect of each moderator, and a table with all statistics obtained for each partition (*Q*_*H*_, *I*^*2*^, and the integrated effect size), as well as the species that are included in each subset.
(DOCX)
