# Supplementary material for: Introducing Meta-Partition, a Useful Methodology to Explore Factors That Influence Ecological Effect Sizes
Source: PLoS One. 2016 Jul 13;11(7):e0158624. doi: 10.1371/journal.pone.0158624 (PMC4943597; doi:10.1371/journal.pone.0158624)
Supplement: S1 Appendix — It comprises the detailed trees of the meta-partition, with comments about the intensity and direction of the effect of each moderator, and a table with all statistics obtained for each partition (QH, I2, and the integrated effect size), as well as the species that are included in each subset. (DOCX) [file pone.0158624.s001.docx]

**S1 APPENDIX: EXTENDED RESULTS OF THE META-PARTITION ABOUT THE SENSITIVITY OF VERTEBRATES TO WETLAND HABITAT LOSS**

**Introductive remarks**

Our re-analysis of the meta-analysis published by Quesnelle et al. (2014)^[[1]](#footnote-1)^ takes into analysis the following moderators that may influence the effect size (correlation between wetland amount and population abundance):

1. Moderators related with the design of the study:
   1. The *study type*: amount vs. configuration
   2. The *sampling effort*: independent / dependent / unknown
   3. The effect of including the study area in the amount of wetland (*patch area*): yes / no
2. Biological traits that may influence sensitivity to wetland habitat loss:
   1. *Home range* (continuous)
   2. *Body length* (continuous)
   3. *Reproductive rate* (continuous)
   4. *Taxon*: amphibians / reptiles / birds / mammals

We include the 334 populations for which there are data for the seven moderators. Data of *home range*, *body length* and *reproductive rate* are all missing for one population (*Podilymbus podiceps* of the study nº 33, see Quesnelle et al., 2014). In addition, there is a population of a bird (*Cistothorus palustris* of the study nº 33, see Quesnelle et al., 2014) for which body length must be misleaded, because Quesnelle et al. (2014) used a length of 122 cm and it must be 12.2 cm. However, we have included both populations in our meta-partition, as they were in the data of Quesnelle et al. (2014), in order to be as comparative as possible with their meta-regressions.

**RESULTS FROM META-PARTITION**

The initial group of 334 populations is highly heterogeneous (Q_H_ = 711.50; p < 0.0001) and the first partition is by *taxon*, in two subsets:

- Amphibians and reptiles, with 213 populations, with mean *z* = 0.074 (sd = 1.45). Thus, amphibians and reptiles would be less sensitive to wetland loss.
- Birds and mammals, with 120 populations, with mean *z* = 0.210 (sd = 1.28). Thus, birds and mammals would be more sensitive to wetland loss.

**S1.1. Amphibians and reptiles**

The subset of amphibians and reptiles includes 213 populations and is highly heterogeneous. First partition is by *study type*, with 2 subsets:

- Amphibians and reptiles studied by *amount*, with 151 populations and mean *z* = 0.058 (sd = 1.37).
- Amphibians and reptiles studied by *configuration*, with 62 populations and mean *z* = 0.132 (sd = 1.58).

Thus, *study type* is the moderator that mainly influences sensitivity to wetland loss among amphibians and reptiles, being significantly more important than the taxon (amphibian vs reptile), the mobility of species or the reproduction rate of species. Specifically, the studies about amphibians and reptiles where wetland amount was calculated as the % wetland area in a landscape or buffer surrounding the sampled area, show smaller effect sizes (less sensitivity to habitat loss) than the studies where the configuration of wetland habitat was included in the calculation of the measure (see Quesnelle et al. 2014 for more details about the study designs). Hence, we should consider the studies conducted by *amount* and by *configuration* in different subsets if we want to assess the hypothesis of mobility or reproductive rate influencing sensitivity to wetland loss in the herpetofauna.

**S1.1.1. Amphibians and reptiles studied by *amount-based* designs**

Within the amphibians and reptiles studied by the *amount* design, the next moderator that affects sensitivity to habitat loss is *sampling effort*, with two new subsets:

- Amphibians and reptiles studied by *amount* and with *sampling effort* being *independent* or *unknown*, with 81 populations and mean *z* = 0.037 (sd = 1.33).
- Amphibians and reptiles studied by *amount* and with *sampling effort* being *dependent*, with 70 populations and mean *z* = 0.100 (sd = 1.36).

Thus, not only the *study type* influences sensitivity to habitat loss in amphibians and reptiles, but for the subset studied by *amount*, the next main moderator is *sampling effort*, being more important that any biological moderator. Therefore, the *amount* studies of amphibians and reptiles on which *sampling effort* was not related to effect size or this factor was unknown show less sensitivity to habitat loss than the studies on which sampling effort was related to the effect size.

**S1.1.1.1. Amphibians and reptiles studied by *amount-based* designs and with *sampling effort* being *independent* of effect size or *unknown***

With the amphibians and reptiles studied by *amount* and with *sampling effort* being *independent* or *unknown* of the effect sizes, the next main moderator that affects sensitivity to wetland habitat loss is the *reproductive rate*, with four new subsets:

- Amphibians and reptiles studied by *amount* and with *sampling effort* being *independent* or *unknown*, and *reproductive rate* of < 910, with 43 populations and mean *z* = 0.005 (sd = 1.35).
- Amphibians and reptiles studied by *amount* and with *sampling effort* being *independent* or *unknown*, and *reproductive rate* of ≥ 3500, with 14 populations and mean *z* = -0.048 (sd = 1.10). This subset is final (**subset 5**) and homogeneous and comprises the following populations: 6 populations of *Bufo americanus, Bufo boreas, Bufo calamita, Bufo cognatus, Bufo fowleri,* 2 populations of *Limnodynastes dumerilii,* and 2 populations of *Rana catesbeiana.*
- Amphibians and reptiles studied by *amount* and with *sampling effort* being *independent* or *unknown*, and *reproductive rate* between 1200 and 3500, with 18 populations and mean *z* = 0.079 (sd = 0.90). This subset is final (**subset 6**) and homogeneous: *Rana clamitans, Rana ridibunda, Rana clamitans,* 4 populations of *Hyla versicolor, Hyla versicolor/H, chrysoscelis, Rana temporaria, Rana arvalis, Bufo bufo,* and 7 populations of *Rana pipiens*.
- Amphibians and reptiles studied by *amount* and with *sampling effort* being *independent* or *unknown*, and *reproductive rate* of < 1200, with 6 populations and mean *z* = 0.157 (sd = 1.41). This subset is final (**subset 7**) and homogeneous: 3 populations of *Pseudacris triseriata, Rana dalmatina,* and 2 populations of *Hyla arborea*.

Empirical evidence is mixed here, suggesting an interaction between the reproductive rate of amphibians and reptiles and their sensitivity to wetland loss. It seems that animals with small and large reproductive rates would be apparently less sensitive to habitat loss regarding its population abundance than populations with middle values of reproductive rates, but more work would be needed to explain the relation.

Within amphibians and reptiles studied by *amount* and with *sampling effort* being *independent* or *unknown*, and *reproductive rate* of < 910, next main moderator is *home range*, with three new subsets:

- Amphibians and reptiles studied by *amount* and with *sampling effort* being *independent* or *unknown*, a *reproductive rate* of < 910, and a *home range* between 6.2 and 20.82 ha, with 5 populations and mean *z* = -0.147 (sd = 0.46). This subset is final (**subset 1**) and homogeneous and comprises the following populations: *Pseudacris maculata, Rana luteiventris, Chelydra serpentina serpentina, Emydoidea blandingii,* and *Sternotherus odoratus.*
- Amphibians and reptiles studied by *amount* and with *sampling effort* being *independent* or *unknown*, a *reproductive rate* of < 910, and a *home range* of < 6.2 ha, and mean *z* = 0.004 (sd = 1.33).
- Amphibians and reptiles studied by *amount* and with *sampling effort* being *independent* or *unknown*, a *reproductive rate* of < 910, and a *home range* of ≥ 20.82 ha, and mean *z* = 0.133 (sd = 0.69). This subset is final (**subset 4**) and homogeneous and comprises the following populations: *Triturus cristatus, Apalone spinifera spinifera, Chrysemys picta marginata, Nerodia erythrogaster neglecta,* and *Trachemys scripta elegans*.

Within amphibians and reptiles studied by *amount* and with *sampling effort* being *independent* or *unknown*, a *reproductive rate* of < 910, and a *home range* of < 6.2 ha, next moderator influencing sensitivity to habitat loss is *body length*, with two new subsets:

- Amphibians and reptiles studied by *amount* and with *sampling effort* being *independent* or *unknown*, a *reproductive rate* of < 910, a *home range* of < 6.2 ha, and a *body length* of < 99 cm, with 32 populations (mean *z* = -0.002, sd = 1.29; **subset 2**) and heterogeneous and comprises the following populations: *Acris crepitans, Ambystoma macrodactylum, Ambystoma maculatum, Ambystoma tigrinum, Ambystoma tigrinum tigrinum, Bombina variegata,* 2 populations of *Crinia signifera, Ichthyosaura alpestris,* 2 populations of *Limnodynastes peronei, Lissotriton helveticus, Lissotriton vulgaris, Paracrinia haswelli,* 4 populations of *Pseudacris crucifer, Pseudacris regilla,* 5 populations of *Rana sylvatica, Rana virgatipes,* 2 populations of *Chelydra serpentina,* 3 populations of *Chrysemys picta, Glyptemys muhlenbergii,* and *Nerodia sipedon pleuralis*.
- Amphibians and reptiles studied by *amount* and with *sampling effort* being *independent* or *unknown*, a *reproductive rate* of < 910, a *home range* of < 6.2 ha, and a *body length* of ≥ 99 cm, with 1 population (*z* = 0.266; **subset 3**) of *Nerodia rhombifer rhombifer*.

**S1.1.1.1. Amphibians and reptiles studied by *amount-based* designs and with *sampling effort* being *dependent* of effect sizes.**

Within the 70 populations of amphibians and reptiles studied by *amount-based* designs and with *sampling effort* being *dependent* of effect sizes, next main moderator that influences sensitivity to wetland loss is, again, the *reproductive rate*, with two new subsets:

- Amphibians and reptiles studied by *amount-based* designs, with *sampling effort* being *dependent*, and *reproductive rates* of ≥ 72.5, with 66 populations and mean *z* = 0.093 (sd = 1.31).
- Amphibians and reptiles studied by *amount-based* designs, with *sampling effort* being *dependent*, and *reproductive rates* of < 72.5, with 4 populations and mean *z* = 0.432 (sd = 1.70).

Within amphibians and reptiles studied by *amount-based* designs, with *sampling effort* being *dependent*, and *reproductive rates* of ≥ 72.5, next main moderator is *body length*, with three new subsets:

- Amphibians and reptiles studied by *amount-based* designs, with *sampling effort* being *dependent*, *reproductive rates* of ≥ 72.5, and *body length* of ≥ 10 cm, with 19 populations and mean *z* = 0.028 (sd = 1.44). (**subset 8**) and heterogeneous and comprises the following populations: 2 populations of *Ambystoma laterale, Ambystoma laterale/A, maculatum,* 5 populations of *Ambystoma maculatum, Ambystoma tigrinum mavortium,* 2 populations of *Bufo bufo,* 4 populations of *Rana catesbeiana,* 2 populations of *Rana temporaria,* and 2 populations of *Triturus cristatus.*
- Amphibians and reptiles studied by *amount-based* designs, with *sampling effort* being *dependent*, *reproductive rates* of ≥ 72.5, and *body length* between 4.1 and 10 cm, with 39 populations (**subset 9**) and mean *z* = 0.102 (sd = 1.25), it is heterogeneous and comprises the following populations: 2 populations of *Bombina variegata,* 2 populations of *Bufo americanus, Bufo cognatus, Bufo fowleri,* 2 populations of *Hyla arborea, Limnodynastes dumerilii, Limnodynastes peronei,* 2 populations of *Lissotriton vulgaris, Litoria raniformis,* 5 populations of *Notophthalmus viridescens, Pelobates fuscus,* 6 populations of *Rana clamitans,* 2 populations of *Rana dalmatina,* 2 populations of *Rana esculenta, Rana muscosa, Rana pipiens,* 6 populations of *Rana sylvatica, Rana utricularia,* and *Spea bombifrons.*
- Amphibians and reptiles studied by *amount-based* designs, with *sampling effort* being *dependent*, *reproductive rates* of ≥ 72.5, and *body length* of < 4.1 cm, with 8 populations (**subset 10**) and mean *z* = 0.225 (sd = 0.99), it is an homogeneous subset and comprises the following populations: *Acris crepitans, Crinia signífera, Hyla versicolor, Hyla versicolor/chrysoscelis,* 3 populations of *Pseudacris crucifer,* and *Pseudacris triseriata.*

This result suggests a possible tradeoff between reproductive rate and mobility in amphibians: small animals (< 4.1 cm) would be highly sensitive to wetland habitat loss although their reproductive rates are relatively high (≥ 72.5).

Within amphibians and reptiles studied by *amount-based* designs, with *sampling effort* being *dependent*, and *reproductive rates* of < 72.5, next main moderator was, again, *body length*, with two new subsets:

- Amphibians and reptiles studied by *amount-based* designs, with *sampling effort* being *dependent*, *reproductive rates* of < 72.5, and *body length* of < 20 cm, with two populations (**subset 11**) and mean *z* = -0.081 (sd = 1.18), it is homogeneous and comprises the following populations: *Chrysemys picta marginata,* and *Emydoidea blandingii.*
- Amphibians and reptiles studied by *amount-based* designs, with *sampling effort* being *dependent*, *reproductive rates* of < 72.5, and *body length* of ≥ 20 cm, with two popularions (**subset 12**) and mean *z* = 0.611 (sd = 0.43), it is homogeneous and comprises the following populations: *Salamandra salamandra,* and *Clemmys guttata.*

Although sample sizes are too small here to be safe from confounding factors, results would be coherent with the tradeoff between reproductive rate and body length mentioned above. At least, results suggest that *Salamandra salamandra, Clemmys guttata* are especially sensitive to wetland habitat loss.

**S1.1.2. Amphibians and reptiles studied by *configuration-based* designs**

Within the amphibians and reptiles studied by the *configuration* design, the next moderator that affects sensitivity to habitat loss is *home range*, with three new subsets:

- Amphibians and reptiles studied by the *configuration* design with a *home range* between 29.1 and 95 ha, with 6 populations and mean *z* = -0.243 (sd = 1.18).
- Amphibians and reptiles studied by the *configuration* design with a *home range* of ≥ 95 ha, with 3 populations mean *z* = 0.154 (sd = 0.91). This subset is final (**subset 15**) and homogeneous: *Rana blairi/R, sphenocephalus, Rana draytonii, Rana pipiens.*
- Amphibians and reptiles studied by the *configuration* design with a *home range* of < 29.1 ha, with 53 populations and mean *z* = 0.161 (sd = 1.47).

Within amphibians and reptiles studied by the *configuration* design with a *home range* between 29.1 and 95 ha, next main moderator affecting habitat loss is, once again, the *reproductive rate*, with two new subsets:

- Amphibians and reptiles studied by the *configuration* design with a *home range* between 29.1 and 95 ha and a *reproductive rate* of < 8000, with 5 populations and mean *z* = -0.314 (sd = 0.43). (**subset 13**), it is homogeneous and comprises the following populations: *Bufo americanus,*  2 populations of *Bufo bufo, Rana sylvatica,* and *Emydoidea blandingii.*
- Amphibians and reptiles studied by the *configuration* design with a *home range* between 29.1 and 95 ha and a *reproductive rate* of ≥ 8000, with 1 population of *Bufo americanus* (**subset 14**) with a mean *z* = 0.172.

Within amphibians and reptiles studied by the *configuration* design with a *home range* of < 29.1 ha, next main moderator affecting habitat loss is, once again, the *reproductive rate*, with two new subsets:

- Amphibians and reptiles studied by the *configuration* design with a *home range* of < 29.1 ha and a *reproductive rate* of < 1100, with 34 populations with mean *z* = 0.106 (sd = 1.16).
- Amphibians and reptiles studied by the *configuration* design with a *home range* of < 29.1 ha and a *reproductive rate* of ≥ 1100, with 19 populations with mean *z* = 0.276 (sd = 1.67). (**subset 19**), it is heterogeneous and comprises the following populations: *Bufo americanus/B, woodhouseii fowleri, Bufo calamita,* 3 populations of *Hyla arborea,* 2 populations of *Hyla versicolor, Hyla versicolor/H, chrysoscelis, Litoria raniformis,* 3 populations of *Rana catesbeiana,* 3 populations of *Rana clamitans, Rana esculenta, Rana lessonae, Rana temporaria,* and *Scinax fuscovarius.*

Within amphibians and reptiles studied by the *configuration* design with a *home range* of < 29.1 ha and a *reproductive rate* of < 1100, next main moderator affecting habitat loss is, once again, the *taxon*, with two new subsets:

- Amphibians studied by the *configuration* design with a *home range* of < 29.1 ha and a *reproductive rate* of < 1100, with 29 populations and mean *z* = 0.087 (sd = 1.12).
- Reptiles studied by the *configuration* design with a *home range* of < 29.1 ha and a *reproductive rate* of < 1100, with 5 populations and mean *z* = 0.336 (sd = 0.60). (**subset 18**), it is homogeneous and comprises the following populations: *Chelodina longicollis, Chrysemys picta, Chrysemys picta marginata, Nerodia erythrogaster neglecta,* and *Nerodia sipedon sipedon.*

Finally, within amphibians studied by the *configuration* design with a *home range* of < 29.1 ha and a *reproductive rate* of < 1100, next main moderator affecting habitat loss is *sampling effort*, with two new subsets:

- Amphibians studied by the *configuration* design with a *home range* of < 29.1 ha, a *reproductive rate* of < 1100 and *sampling effort* dependent, with 23 populations and mean *z* = 0.063 (sd = 1.08). (**subset 16**), it is homogeneous and comprises the following populations: *Acris crepitans, Ambystoma maculatum, Ambystoma maculatum/A, jeffersonianum, Ambystoma texanum, Ambystoma tigrinum tigrinum, Hemidactylium scutatum,* 2 populations of *Ichthyosaura alpestris, Lissotriton helveticus,* 2 populations of *Lissotriton vulgaris,* 2 populations of *Notophthalmus viridescens,* 4 populations of *Pseudacris crucifer, Pseudacris maculate*, 2 populations of *Pseudacris triseriata,* 2 populations of *Rana sylvatica,* and *Triturus cristatus.*
- Amphibians studied by the *configuration* design with a *home range* of < 29.1 ha, a *reproductive rate* of < 1100 and *sampling effort* independent or unknown, with 6 populations and mean *z* = 0.179 (sd = 1.05; **subset 17**), it is homogeneous and comprises the following populations: *Engystomops pustulosus,* 2 populations of *Ichthyosaura alpestris, Lissotriton helveticus, Pseudacris regilla,* and *Triturus cristatus.*

**S1.2. Birds and mammals**

The subset of birds and mammals includes 121 populations and is highly heterogeneous (DATO). First partition is again by *taxon*, with 2 subsets:

- Birds, with 115 populations and smaller effect sizes (mean *z* = 0.185 ± 1.21).
- Mammals, with 6 populations and larger effect sizes (mean *z* = 0.408 ± 0.94).

Thus, wetland mammals are much sensitive to habitat loss than birds.

**S1.2.1. Birds**

Within birds, the main trait influencing sensitivity to habitat loss is *body length*, with two subsets:

- Birds with a length < 94 cm, with 104 populations and smaller effect sizes (mean *z* = 0.165 ± 1.12).
- Birds with a length ≥ 94 cm, with 11 populations and larger effect sizes (mean *z* = 0.511 ± 0.98). This subset is final and homogeneous (**subset 29**), and comprises the following populations: *Ardea alba, Ardea cinérea,* 3 populations of *Ardea herodia,* 4 populations of *Cistothorus palustris. Grus canadensis,* and *Xanthocephalus xanthocephalus.* Here it is necessary to warn that *Cistothorus palustris* is a little bird of about 10 cm and in the data base of Quesnelle et al. (2014) it is wrongly assigned a length of 122 cm. We kept the data in our analysis in order to replicate it and compare it with the meta-regressions of Quesnelle et al. (2014).

Thus, the main factor that influences sensitivity to habitat loss in birds is body length (related to mobility), with a positive relationship: bigger birds (≥ 94 cm) are much more sensible to habitat loss.

Within birds smaller than 94 cm, the next main trait affecting sensitivity to habitat loss is the *reproductive rate*, with two new subsets:

- Birds with a length < 94 cm and a reproductive rate of ≥ 3.9, with 84 populations and mean *z* = 0.142 (sd = 1.01).
- Birds with a length < 94 cm and a reproductive rate of < 3.9, with 20 populations and *z* = 0.288 (sd = 1.33).

Thus, among birds smaller than 94 cm, reproductive rate is the most important trait, and is negatively related to sensitivity to habitat loss.

Within birds that are smaller than 94 cm and have a reproductive rate of ≥ 3.9, the next main trait that affects habitat loss is *patch area*, with two new subsets:

- Birds with a length < 94 cm, a reproductive rate of ≥ 3.9 and a *patch area* NO, with 24 populations and mean *z* = 0.104 (sd = 0.77).
- Birds with a length < 94 cm, a reproductive rate of ≥ 3.9 and a *patch area* YES, with 60 populations and mean *z* = 0.149 (sd = 1.09).

Within birds that are smaller than 94 cm, have a reproductive rate of ≥ 3.9 and a *patch area* NO, the next main trait that affects habitat loss is *home range*, with two new subsets:

- Birds with a length < 94 cm, a reproductive rate of ≥ 3.9, *patch area* NO, and a *home range* of < 1.22 ha, with 11 populations and mean *z* = 0.06 (sd = 0.93)*.*
- Birds with a length < 94 cm, a reproductive rate of ≥ 3.9, *patch area* NO, and a *home range* of ≥ 1.22 ha, with 13 populations and mean *z* = 0.256 (sd = 0.43)*.* This subset (**subset 22)** is final and homogeneous, and comprises the following populations: *Botaurus lentiginosus, Cettia cetti,* 2 populations of *Circus aeruginosus, Circus cyaneus, Gallinago delicata,* 2 populations of *Gallinula galeata, Ixobrychus exilis, Podilymbus podiceps, Rallus elegans, Remiz pendulinus,* and *Tachybaptus ruficollis.*

Within birds with a length < 94 cm, a reproductive rate of ≥ 3.9, *patch area* NO, and a *home range* of < 1.22 ha, the next main trait affecting sensitivity to habitat loss the *study type*, with two subsets:

- Within birds with a length < 94 cm, a reproductive rate of ≥ 3.9, *patch area* NO, a *home range* of < 1.22 ha, and studied by *configuration*, with 5 populations and mean *z* = -0.222 (sd = 0.47). This group is final (**subset 20**) and homogeneous, and comprises the following populations: *Acrocephalus arundinaceus, Acrocephalus scirpaceus, Porzana Carolina, Rallus aquaticus,* and *Rallus limicola.*
- Within birds with a length < 94 cm, a reproductive rate of ≥ 3.9, *patch area* NO, a *home range* of < 1.22 ha, and studied by *amount*, with 6 populations and mean *z* = 0.096 (sd = 0.92). This is a final subset (**subset 21**), and homogeneous, and comprises the following populations: *Acrocephalus scirpaceus, Agelaius phoeniceus, Geothlypis trichas, Porphyrio martinica, Porzana Carolina,* and *Rallus limicola.*

**S1.2.2. Mammals**

Although sample size of mammals is very small (n = 6), we make one partition with explorative intentions. Thus, within mammals, the main trait influencing sensitivity to habitat loss is the *study type*, with two subsets:

- Mammals studied by *amount*, with 5 populations and mean *z* = 0.247 (sd = 0.43). This is a final and homogeneous subset (**subset 30**) and comprises the following populations: *Castor Canadensis, Microtus pennsylvanicus, Oryzomys palustris, Sorex fumeus, Sylvilagus palustris hefneri.*
- Mammals studied by *configuration*, with 1 population of *Neofiber alleni* (**subset 31**) with *z* = 0.448.

**Figure A.** Summary tree of the meta-partition of amphibians and reptiles. Within each partition, effect sizes are organized from left (smaller) to right (larger). The best moderator that explains the variability of each partition is reported into a grey arrow, and numbers in brackets means (in the form of *X*/*Y*, e.g. 9/10) the number of times this partition is kept in the sensitivity analysis (*X*) in comparison with the number of times that is partition is replicated (*Y*).


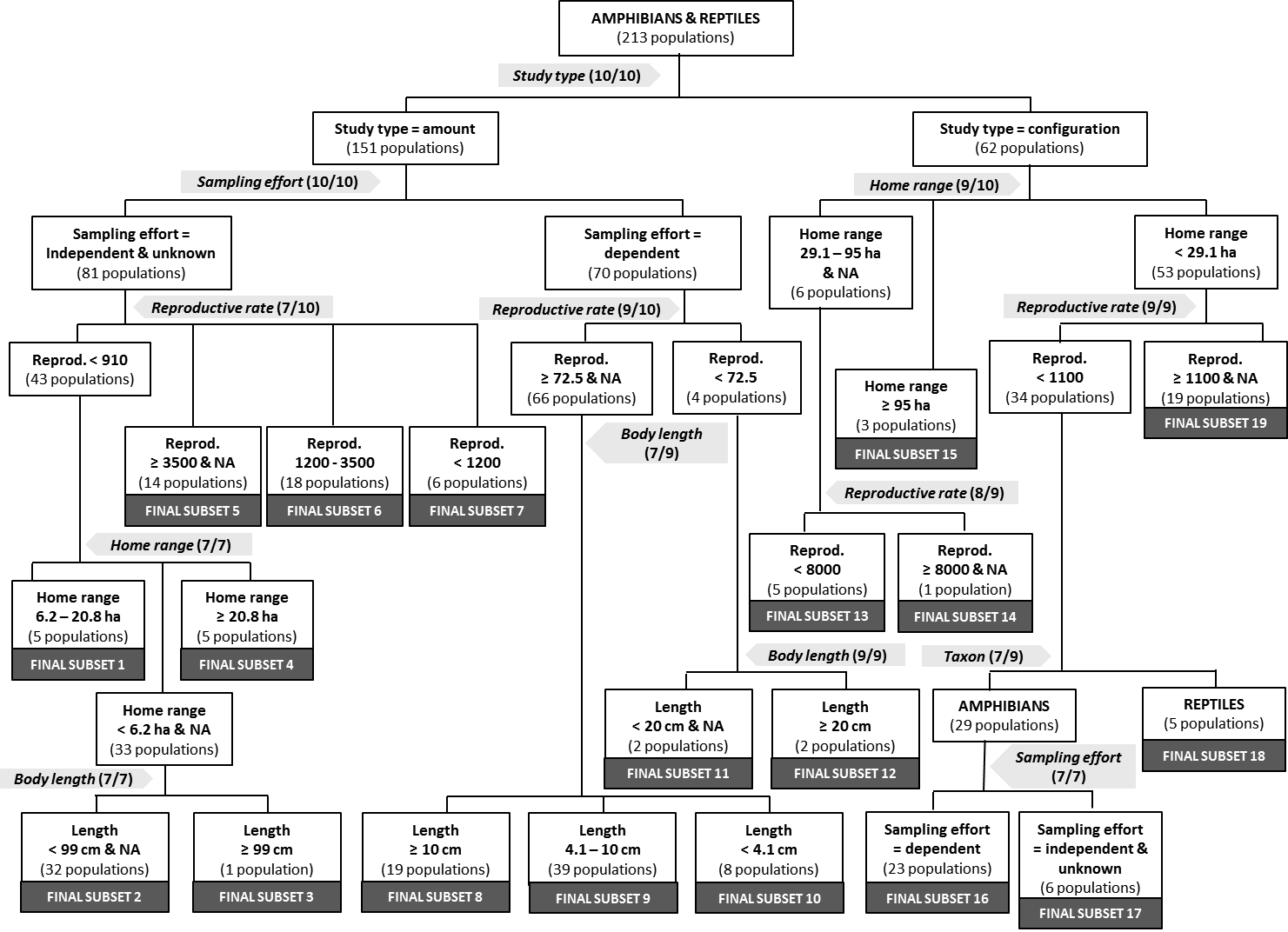


**Figure B.** Summary tree of the meta-partition of birds and mammals. Within each partition, effect sizes are organized from left (smaller) to right (larger). The best moderator that explains the variability of each partition is reported into a grey arrow, and numbers in brackets means (in the form of *X*/*Y*, e.g. 9/10) the number of times this partition is kept in the sensitivity analysis (*X*) in comparison with the number of times that is partition is replicated (*Y*).


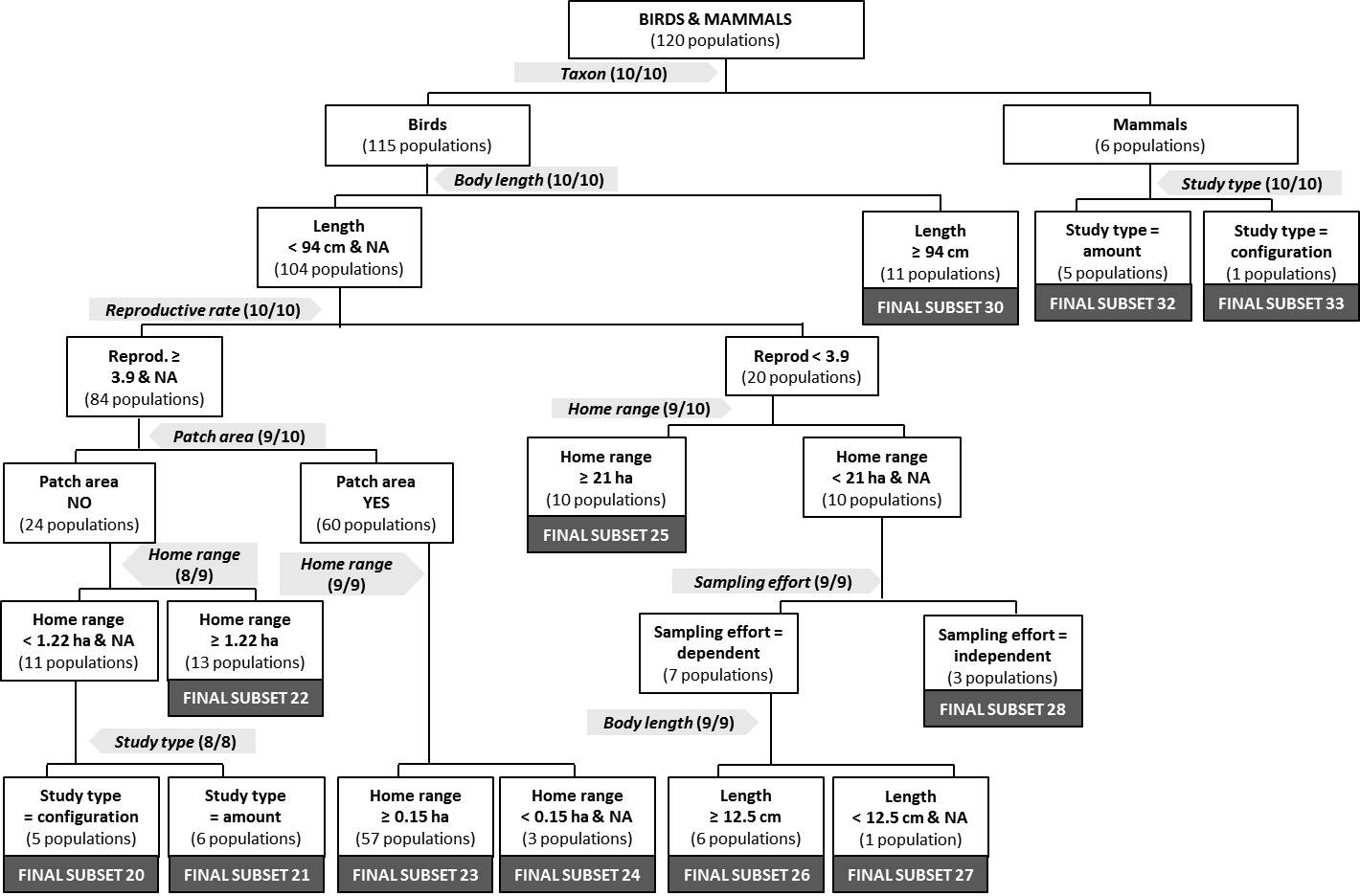


**Table A. Results of meta-partition of the sensitivity to wetland habitat loss by moderators related with the design of the study and biological moderators.** There are 33 final subsets that result from the meta-partition, the number of each subset is given in the first column, with the number of populations included in the subsets in brackets. The effect size is integrated by the fixed effect model in the homogeneous subsets (p-value of the test of homogeneity > 0.05). If the subset is heterogeneous, effect sizes are integrated with the random effects model (marked with a *). The confidence interval (CI) provided to the effect sizes (ES_r_) is the 95% CI.

| **Subset**  **(n)** | **Q_H_**  **(p-value)** | **I^2^** | **Integrated ES_r_ (CI)** | **Populations included** |
| --- | --- | --- | --- | --- |
| 1 (5) | 0.835  (0.934) | 0 | -0.146  (-0.243 / -0.047) | *Pseudacris maculata, Rana luteiventris, Chelydra serpentina serpentina, Emydoidea blandingii,* and *Sternotherus odoratus* |
| 2 (32) | 51.469  (0.012) | 39.77 % | -0.000  (-0.055 / 0.054)* | *Acris crepitans, Ambystoma macrodactylum, Ambystoma maculatum, Ambystoma tigrinum, Ambystoma tigrinum tigrinum, Bombina variegata,* 2 populations of *Crinia signifera, Ichthyosaura alpestris,* 2 populations of *Limnodynastes peronei, Lissotriton helveticus, Lissotriton vulgaris, Paracrinia haswelli,* 4 populations of *Pseudacris crucifer, Pseudacris regilla,* 5 populations of *Rana sylvatica, Rana virgatipes,* 2 populations of *Chelydra serpentina,* 3 populations of *Chrysemys picta, Glyptemys muhlenbergii,* and *Nerodia sipedon pleuralis* |
| 3 (1) |  |  | 0.266 | *Nerodia rhombifer rhombifer* |
| 4 (5) | 1.921  (0.750) | 0 | 0.132  (0.044 / 0.218) | *Triturus cristatus, Apalone spinifera spinifera, Chrysemys picta marginata, Nerodia erythrogaster neglecta,* and *Trachemys scripta elegans* |
| 5 (14) | 15.656  (0.268) | 16.96 | -0.048  (-0.122 / 0.027) | 6 populations of *Bufo americanus, Bufo boreas, Bufo calamita, Bufo cognatus, Bufo fowleri,* 2 populations of *Limnodynastes dumerilii,* and 2 populations of *Rana catesbeiana* |
| 6 (18) | 13.846  (0.678) | 0 | 0.079  (0.028 / 0.129) | *Rana clamitans, Rana ridibunda, Rana clamitans,* 4 populations of *Hyla versicolor, Hyla versicolor/H, chrysoscelis, Rana temporaria, Rana arvalis, Bufo bufo,* and 7 populations of *Rana pipiens* |
| 7 (6) | 9.884  (0.078) | 49.41 % | 0.155  (0.095 / 0.215) | 3 populations of *Pseudacris triseriata, Rana dalmatina,* and 2 populations of *Hyla arborea* |
| 8 (19) | 37.506  (0.004) | 52.01 % | 0.025  (-0.077 / 0.125)* | 2 populations of *Ambystoma laterale, Ambystoma laterale/A, maculatum,* 5 populations of *Ambystoma maculatum, Ambystoma tigrinum mavortium,* 2 populations of *Bufo bufo,* 4 populations of *Rana catesbeiana,* 2 populations of *Rana temporaria,* and 2 populations of *Triturus cristatus* |
| 9 (39) | 59.140  (0.015) | 35.75 % | 0.092  (0.032 / 0.151)* | 2 populations of *Bombina variegata,* 2 populations of *Bufo americanus, Bufo cognatus, Bufo fowleri,* 2 populations of *Hyla arborea, Limnodynastes dumerilii, Limnodynastes peronei,* 2 populations of *Lissotriton vulgaris, Litoria raniformis,* 5 populations of *Notophthalmus viridescens, Pelobates fuscus,* 6 populations of *Rana clamitans,* 2 populations of *Rana dalmatina,* 2 populations of *Rana esculenta, Rana muscosa, Rana pipiens,* 6 populations of *Rana sylvatica, Rana utricularia,* and *Spea bombifrons* |
| 10 (8) | 6.863  (0.443) | 0 | 0.222  (0.097 / 0.339) | *Acris crepitans, Crinia signífera, Hyla versicolor, Hyla versicolor/chrysoscelis,* 3 populations of *Pseudacris crucifer,* and *Pseudacris triseriata* |
| 11 (2) | 1.402  (0.236) | 28.70 % | -0.081  (-0.477 / 0.343) | *Chrysemys picta marginata,* and *Emydoidea blandingii* |
| 12 (2) | 0.184  (0.668) | 0 | 0.545  (0.338 / 0.702) | *Salamandra salamandra,* and *Clemmys guttata* |
| 13 (5) | 0.744  (0.946) | 0 | -0.304  (-0.429 / -0.167) | *Bufo americanus,*  2 populations of *Bufo bufo, Rana sylvatica,* and *Emydoidea blandingii* |
| 14 (1) |  |  | 0.17 | *Bufo americanus* |
| 15 (3) | 1.674  (0.433) | 0 | 0.153  (-0.059 / 0.351) | *Rana blairi/R, sphenocephalus, Rana draytonii, Rana pipiens* |
| 16 (23) | 25.748  (0.263) | 14.56 % | 0.063  (0.010 / 0.116) | *Acris crepitans, Ambystoma maculatum*  *Ambystoma maculatum/A, jeffersonianum, Ambystoma texanum, Ambystoma tigrinum tigrinum, Hemidactylium scutatum,* 2 populations of *Ichthyosaura alpestris, Lissotriton helveticus,* 2 populations of *Lissotriton vulgaris,* 2 populations of *Notophthalmus viridescens,* 4 populations of *Pseudacris crucifer, Pseudacris maculate,* 2 populations of *Pseudacris triseriata,* 2 populations of *Rana sylvatica,* and *Triturus cristatus* |
| 17 (6) | 5.485  (0.359) | 8.85 % | 0.177  (0.074 / 0.277) | *Engystomops pustulosus,* 2 populations of *Ichthyosaura alpestris, Lissotriton helveticus, Pseudacris regilla,* and *Triturus cristatus* |
| 18 (5) | 1.441  (0.837) | 0 | 0.325  (0.168 / 0.465) | *Chelodina longicollis, Chrysemys picta, Chrysemys picta marginata, Nerodia erythrogaster neglecta,* and *Nerodia sipedon sipedon* |
| 19 (19) | 50.423  (<0.001) | 64.30 % | 0.220  (0.108 / 0.326) | *Bufo americanus/B, woodhouseii fowleri, Bufo calamita,* 3 populations of *Hyla arborea,* 2 populations of *Hyla versicolor, Hyla versicolor/H, chrysoscelis, Litoria raniformis,* 3 populations of *Rana catesbeiana,* 3 populations of *Rana clamitans, Rana esculenta, Rana lessonae, Rana temporaria,* and *Scinax fuscovarius* |
| 20 (5) | 0.875  (0.928) | 0 | -0.218  (-0.493 / 0.095) | *Acrocephalus arundinaceus, Acrocephalus scirpaceus, Porzana Carolina, Rallus aquaticus,* and *Rallus limicola* |
| 21 (6) | 4.244  (0.515) | 0 | 0.096  (0.006 / 0.197) | *Acrocephalus scirpaceus, Agelaius phoeniceus, Geothlypis trichas, Porphyrio martinica, Porzana Carolina,* and *Rallus limicola* |
| 22 (13) | 2.191  (0.999) | 0 | 0.251  (0.059 / 0.424) | *Botaurus lentiginosus, Cettia cetti,* 2 populations of *Circus aeruginosus, Circus cyaneus, Gallinago delicata,* 2 populations of *Gallinula galeata, Ixobrychus exilis, Podilymbus podiceps, Rallus elegans, Remiz pendulinus,* and *Tachybaptus ruficollis* |
| 23 (57) | 62.326  (0.292) | 8.54 % | 0.139  (0.104 / 0.175) | 2 populations of *Agelaius phoeniceus, Anas discors, Anas platyrhynchos, Aramus guarauna, Ardeola ralloides, Arenaria interpres,* 4 populations of *Botaurus lentiginosus, Botaurus stellaris, Bubulcus ibis, Calidris alba, Calidris alpine, Charadrius alexandrines, Circus cyaneus,* 2 populations of *Cistothorus platensis, Egretta garzetta, Egretta intermedia,* 2 populations of *Fulica Americana, Gallinago delicate, Gallinula galeata, Geothlypis trichas, Himantopus himantopus,* 2 populations of *Ixobrychus exilis, Limosa fedoa, Limosa lapponica, Numenius arquata, Numenius madagascariensis,* 2 populations of *Nycticorax nycticorax, Pluvialis fulva, Pluvialis squatarola,* 6 populations of *Podilymbus podiceps, Porphyrio martinica,* 4 populations of *Porzana Carolina,* 4 populations of *Rallus limicola, Recurvirostra Americana, Rostrhamus sociabilis, Setophaga petechial, Tringa semipalmata, and Tringa totanus* |
| 24 (3) | < 0.0001  (1) | 0 | 0.430  (0.233 / 0.593) | *Jacana jacana, Rollandia rolland* |
| 25 (10) | 3.902  (0.918) | 0 | 0.105  (0.027 / 0.234) | *Ardea alba, Calidris canutus, Chlidonias niger, Egretta thula,* 2 populations of *Ixobrychus exilis, Mycteria americana, Nycticorax nycticorax, Platalea ajaja,* and *Plegadis chihi* |
| 26 (6) | 4.836  (0.436) | 0 | 0.231  (0.111 / 0.345) | *Acrocephalus rufescens, Empidonax alnorum, Ixobrychus exilis,* 2 populations of *Melospiza georgiana,* and *Setophaga petechia* |
| 27 (1) |  |  | 0.650 | *Geothlypis trichas* |
| 28 (3) | 0.684  (0.710) | 0 | 0.484  (0.358 / 0.593) | 2 populations of *Ixobrychus exilis,* and *Melospiza georgiana* |
| 29 (11) | 9.537  (0.482) | 0 | 0.471  (0.369 / 0.561) | *Ardea alba, Ardea cinérea,* 3 populations of *Ardea herodia,* 4 populations of *Cistothorus palustris. Grus Canadensis,* and *Xanthocephalus xanthocephalus* |
| 30 (5) | 0.755  (0.944) | 0 | 0.242  (0.062 / 0.406) | *Castor Canadensis, Microtus pennsylvanicus, Oryzomys palustris, Sorex fumeus, Sylvilagus palustris hefneri* |
| 31 (1) |  |  | 0.420 | *Neofiber alleni* |

1. Quesnelle, P.E., Lindsay, K.E. & Fahrig, L. (2014) Low reproductive rate predicts species sensitivity to habitat loss: a meta-analysis of wetland vertebrates. *PLoS ONE*, 9, e90926. [↑](#footnote-ref-1)
